# Hyperinsulinemic Hypoglycaemia in a Turner Syndrome with Ring (X)

**DOI:** 10.1155/2015/561974

**Published:** 2015-04-29

**Authors:** Michela Cappella, Vanna Graziani, Antonella Pragliola, Alberto Sensi, Khalid Hussain, Claudia Muratori, Federico Marchetti

**Affiliations:** ^1^Department of Paediatrics, Santa Maria delle Croci Hospital, 48121 Ravenna, Italy; ^2^Department of Clinical Pathology, Medical Genetics Unit, Pievesestina, 47522 Cesena, Italy; ^3^London Centre for Pediatric Endocrinology and Metabolism, Great Hormond Street Hospital for Children NHS Trust and the Institute of Child Health, London WC1N 3JH, UK

## Abstract

Hyperinsulinemic hypoglycaemia (HH) is a group of clinically, genetically, and morphologically heterogeneous disorders characterized by dysregulation of insulin secretion by pancreatic beta cells. HH can either be congenital genetic hyperinsulinism or associated with metabolic disorder and syndromic condition. Early identification and meticulous management of these patients is vital to prevent neurological insult. There are only three reported cases of HH associated with a mosaic, r(X) Turner syndrome. We report the four cases of an infant with a mosaic r(X) Turner genotype and HH responsive to diazoxide therapy.

## 1. Introduction

Hyperinsulinaemic hypoglycaemia (HH) is an important cause of hypoglycaemia in childhood based on an unregulated insulin secretion by *β* cells [1]. Early identification and meticulous management of these patients is vital to prevent neurological insult. We report a case of a child with a mosaic genotype of Turner's syndrome in association with otherwise unexplained persistent HH. This association suggests that HH may be another atypical feature of mosaic Turner's syndrome.

## 2. Case Report

We report a case of a girl, born preterm at 32 weeks of gestation by caesarean section due to intrauterine growth retardation and alteration of fetal blood flow. Her birth weight was 1455 g (10th–25th centile), length was 40 cm (10th–25th centile), and head circumference was 27 cm (10th). The pregnancy was uneventful and her mother was healthy and nondiabetic. Her family history was unremarkable.

Prenatal ultrasound scans were negative, without any evidence of heart disease. Soon after birth a diagnosis of aortic stenosis with bicuspid aortic valve was made and, at the age of three weeks, she underwent aortic valvuloplasty. Neonatal metabolic screening was normal. No episodes of neonatal hypoglycaemia were documented. After discharge she had no serious illnesses or hospitalization, the cardiologic follow-up was carried out without complications, and she had been growing and developing normally.

At 11 months of age, during her first feed in the morning, she presented an episode of generalized tonic-clonic seizures and staring that lasted five minutes. The episode was not associated with fever or any sign of infection. By the time she arrived at our emergency department, she was a little drowsy, but she could be awakened easily.

Because of her previous history of congenital cardiac defect, we immediately performed an echocardiogram and an electrocardiogram although they did not show any cardiovascular acute disease. Respiratory, abdominal, and neurologic examinations were normal. A complete blood count, C-reactive protein, and electrolyte panel were normal but hypoglycaemia with a blood glucose concentration of 1.8 mmol/l (range 3.3–5.5 mmol/l) has been found. Transaminases and creatine phosphokinase were normal. Acid-base balance and lactate showed no abnormality. Her thyroid function test and cortisol, adrenocorticotropic, and growth hormone were normal. Urine analysis was without ketonuria. The levels of organic acids, amino acids in plasma, acylcarnitines, and free carnitine were normal. Electroencephalogram (EEG) and video-EEG as well did not show any abnormalities. No hepatomegaly or pancreatic alteration resulted from abdominal ultrasound. Brain magnetic resonance imaging showed only a delay in myelination but no major abnormalities.

Hypoglycaemia resolved with continuous infusion of glucoelectrolytic solution, but soon after suspension, her random blood glucose controls showed hypoglycaemic episodes especially in the early morning hours, with blood glucose levels around 1.6–2.5 mmol/l (range 3.3–5.5 mmol/l). At the lower glucose value she presented hypoglycaemic symptoms (pallor, sweating, and motor delay) but not seizures. At this time elevated insulin (7.4 mU/L; range < 2 mU/L) was found accompanied by mild hypoketonemia and hypofattyacidaemic hypoglycaemia (0.3 mmol/l) (Table 1).

Then she was commenced on glucose infusion of 10% dextrose (8 mg/kg/min) and hypoglycaemia was corrected. In addition, a positive glycemic response to glucagon has been proven. Overall, the biochemical pictures were consistent with HH.

The serum ammonia concentration was normal and no mutation was found in the* ABCC8, KCNJ11 GCK, HADH, UCP2, *and* HNF4A *genes.

On physical examination she presented very mild dysmorphic elements: prominent forehead, plagiocephaly, long palpebral fissures, mild synophrys, thin upper lip, mildly short neck, and irregular palmar creases. Her growth parameters were normal (between 25th and 50th centile in weight and length). She showed a very mild motor delay.

Cytogenetic analysis revealed 2 cell lines: 89% showed a single normal X chromosome and a small ring chromosome, 46 X, r(X); 11% had just a single normal X chromosome, 45 X. No 46 XX cells were found out of 100 examined. Fluorescent in situ hybridization (FISH) studies showed that the X inactivation locus (XIST) was present on the ring X. Cytogenetic findings are associated with Turner syndrome (TS).

After four days glucose infusion was progressively stopped and at the same time an introduction of maltodextrin into her food and drink has been started. She still had intermittent low blood glucose levels. Based on the algorithm for hyperinsulinism's treatment she was treated with diazoxide and responded well at dose of 8 mg/kg/day, with stabilized blood glucose levels and a longer fasting tolerance.

At the time of writing, the proband is 26 months old and is able to fast for 12 hours without developing hypoglycaemia on a low dose of diazoxide (4 mg/kg/day).

## 3. Discussion

HH can either be due to congenital hyperinsulinism caused by genetic defects (CHI) in key genes regulating insulin secretion or secondary to certain perinatal risk factors (birth asphyxia, intrauterine growth retardation, and maternal diabetes mellitus) or associated with metabolic conditions (congenital disorders of glycosylation, *β*-oxidation defects). In adults an insulinoma accounts for most cases of HH [1].

Our patient presented at 11 months of age with seizure as the first clinical manifestation of persistent hypoglycaemia and reduced fasting tolerance with no ketonuria. Our work-up to differential diagnosis showed hyperinsulinism (high insulin plasma level, hypoketonaemia, hypofattyacidaemia at lower glucose level, high glucose requirement to maintain normoglycaemia, and positive glycaemic response to glucagon) [1]. The differential diagnosis of *β*-oxidation defects could be excluded because of the normal plasmatic acylcarnitine.

Nowadays the mutations in nine genes are currently known to cause HH. The most common (about 40–70% of cases) and severe types are due to* ABCC8* and* KCJN11* (encoding SUR-1 and Kir 6.2, resp.) in which inactivating mutations in these genes encoding the two subunits of the *β* cell ATP-sensitive potassium channel cause the hyperinsulinism. The effect of these mutations on channel expression and function determines the clinical phenotype, particularly the response to diazoxide [2, 3]. No more common typical mutations in diazoxide-responsive CHI genes were identified. So the response to diazoxide in our patient may suggest that her hyperinsulinaemia is originated from other mechanisms besides disorder of K-ATP channel. The serum ammonia was normal so we ruled out the hyperinsulinism-hyperammonaemia syndrome, the second common form of CHI (caused by mutation in the* GLUD1* gene) [2, 3].

Syndromic forms of persistent hyperinsulinism have been recognized in association with trisomy 13, Beckwith-Wiedemann syndrome, congenital disorders of glycosylation, Perlman syndrome, and Soto's syndrome and in some as yet undiagnosed syndromes [1]. The mechanisms of HH in these syndromes are largely unclear [1].

Our patient had the TS. She showed mild dysmorphic elements, her growth parameters were between the 25th and the 50th percentile in weight and length, and just congenital heart disease was suggestive for TS [4].

In the literature another three cases of TS with HH have been described [5–7]. The first case [5] was an infant, who, after newborn hypoglycemia, was studied at 4, 8, and 10 months for hypoglycemic convulsions and with a subsequent leucine loading test compatible with hyperinsulinism. Her complex karyotype showed a monosomic 45 X cell line and two cell lines with an X chromosome derived ring that was duplicated and dicentric in the less frequent cell line: 46 X,+mar[19]/45 X[8]/46 X,+mar2[31].ish r(X)(DXZI+)[81]/dic r(X) (DXZ1++)[2].

A second reported case [6] was a 4-months-old girl with complex mosaic karyotype with four different cell lines: mos 46 X, r(X)/47 X, r(X) + der(X)/46 X,der(X)/45 X. XIST gene was present on the ring, but not on the der(X). This case was admitted with staring episodes and generalized tonic-clonic seizures associated with low blood glucose levels. She showed the typical findings for hyperinsulinism.

The third case [7] was a 13-month-old girl with mosaic TS and mild “Kabuki-like” phenotype as defined by the authors. The karyotype showed a mosaicism with three cell lines: 46 X,r(X)/47 r(X), dup r(X)/45 X. XIST was not investigated, but G banding was considered indicative for XIST presence. She was admitted two times to the hospital with spells; persistent hypoglycemic episodes were associated with a hyperinsulinism.

Unlike our patient, these cases showed their first hypoglycemic episodes during the first days of life and then became symptomatic again at the age of 4 [5, 7] and 9 months [6]. Between these points, any subclinical hypoglycemic episodes remained possibly undetected. Two cases were well responsive to diazoxide and this treatment stabilized blood glucose levels and a longer fasting tolerance. The mechanism leading to HH in mosaic TS is largely not clear [5–7]. The reduced insulin secretion in young, nonobese females with a normal insulin sensitivity implies that beta cell dysfunction or insufficiency is a primary feature of the Turner metabolic syndrome [8].

Moreover it is known that X monosomy increases the risk of glucose intolerance and subsequent diabetes mellitus [9]. However, according to Gravholt et al. [10] in TS there is just “an insignificant tendency towards hypoglycemia”: in her large survey on 594 TS she found just 2 cases (0.57 expected, relative risk 3.51, IC: 0.43–12.69). Moreover, our search in PubMed looking for “hypoglycemia and Turner Syndrome” just retrieved the three mosaic cases already cited, all with a ring X chromosome [5–7], a chromosomal abnormality that is found just in 16% of TS karyotypes [11].

So, although large epidemiological data about r(X) and hypoglycemia are lacking, an association between r(X) in TS and HH seems likely.

Our patient presents a mosaic karyotype with a preponderant 46 X,r(X) cell line, showing a tiny ring of the chromosome X (XIST positive) replacing a normal X, and a minor component (11%) with X monosomy (45 X). The presence of the XIST gene on the ring chromosome is correlated with its inactivation while the absence of XIST region prevents the abnormal X chromosome from inactivation, leading to a functional disomy of regions of chromosome X associated with a more severe phenotype with mental retardation and facial features resembling those of the monogenic Kabuki syndrome (in particular the suggestive eversion of the lateral part of the lower lid was noted) [12–14]. The correlation of this severe phenotype and XIST absence is not absolute [13].

Ring chromosomes often show some instability and cause a “dynamic mosaicism,” consisting of mitotic rearrangements (mainly due to the consequences of sister chromatid exchanges in a ring chromosome) that lead to tissues chromosomal heterogeneity [15]. In previously reported cases [5–7] this instability was evident in blood, while in our case it was not; nonetheless instability could be relevant in tissues different from the tested lymphocytes.

Instability generates rearranged chromosomes with duplications and deletions and could also involve XIST or its expression in some tissues.

The pathogenetic mechanism by which the ring X might cause HH is unknown and just speculative hypotheses can be proposed [5–7]. Haploinsufficiency of genes involved in the insulin pathway [6] due to deletion in the ring chromosome seems unlikely, because under this hypothesis homogenous 45 X karyotype should be at least equally predisposing to HH.

Another hypothesis [7] is based on the expression of genes located on the ring, accounting for the functional disomy of part of chromosome X with a mechanism like that proposed for the tiny r(X) for the severe phenotype with mental retardation [16]. This mechanism could be considered also for HH, although XIST seems to be present in all the three cases reported and also in the present case.

A third hypothesis that we put out takes into account the selective mechanism that generally brings to skewed X inactivation: when in a female an X chromosome is structurally abnormal lyonization proceeds randomly, but cells inactivating the normal X are subjected to selective elimination and the great majority of them stop and are eliminated from the cell pool [15]. It is conceivable that in some tissue skewing could be incomplete leaving some unbalanced cells with pathogenetic consequences; moreover the selective apoptotic mechanism involved in cell selection during embryonic stages might interfere with development causing specific pathologies.

## 4. Conclusions

The recognition of hypoglycaemia in children with TS is important in order to prevent further brain damage caused by hypoglycaemic episodes and seizures. Untreated, or inappropriately treated, these children may suffer from hypoglycaemic brain damage in addition to their mental retardation.

Although the mechanism leading to hyperinsulinism in this condition is still unknown, the present report is the fourth case of a female with TS with ring chromosome X associated with congenital hyperinsulinism. However, the interpretation of the effect of cells with different karyotypes is difficult without karyotyping the pancreatic *β* cells. Further studies are required to understand whether the mosaic over- or underexpression of unidentified X chromosome gene(s) in the pancreatic cells leads to hyperinsulinaemic hypoglycaemia. Even if the relationship between the presence r(X) and the HH complication in TS has to be proved, it could not only be a casual association.

## Figures and Tables

**Table 1:** Results of the metabolites and hormones measured at the time of hypoglycemia in our child.

Hormone/metabolite	Value	Reference
Plasma glucose (mmol/L)	1.8	3.5–5.5
Serum insulin (mU/L)	7.4	<2
Serum cortisol (mmol/L)	280	<500
Serum growth hormone (mIU/L)	21	>20
Free fatty acids (mmol/L)	0.3	Raised (>0.5)
Total ketone bodies (mmol/L)	0.04 (suppressed)	Raised (>0.6)
Plasma lactate (mmol/L)	1.8	<2
Serum ammonia (*µ*mol/L)	61	<80

## Abbreviations

CHI: Congenital hyperinsulinism caused by genetic defects
EEG: Electroencephalogram
HH: Hyperinsulinemic hypoglycaemia
TS: Turner syndrome.
